# Shams

**Published:** 1866-05

**Authors:** 


### SHAMS.

This is the age of shams, we are met with deceptions at every corner ; that veteran soldier has one of Palmer's legs, and you couldn't tell it from a real one. You have been sitting at the table for a month by the very side of a man who has been eating with Allen's teeth and you never knew any better ; wooden churches are made to represent brown stone ; milk is no longer milk, except Canfield's ; coffee is made out of burnt bread crust ; friends smile most sweetly when they contemplate a fraud, and their very presents are bribes. A new sham has sprung up of late, in high places mostly, which is about as cool a piece of beggary, as any thing we have become acquainted with in the whole course of our natural life. Aristocratic father is "hard up ;" his daughter is about to be married ; he has no portion to give her, while "everybody" was sure that she would have a splend outfit, and for "everybody" to be disappointed would never do ; what a triumph it would be to enemies ; what a mortification to friends ; what a sweet morsel for the malicious. The wind must be raised by hook or crook, and the programme is on this wise, a choice few only being in the secret : The object in the first place is to make an impression intended to advance the social position ; but a more substantial aim is in view, and to be accomplished in a very gentlemanly way. It is given out that the best of that "set" are going to make bridal presents; now, in all sets, there are always crawling apes; persons who seek to be number ones, by imitating them; so they express an intention of doing the same thing; then comes in another class to increase the little army; those who socially are equal to number one, but having been "unfortunate in business" are "very much cramped for want of means;" they are not really able to do as "everybody" does; but the necessity of the case compel them to do something; that something they would like to be very "handsome," and being poor and proud they are in a most perplexing quandary, but pride becomes the victor and a "present" is decided upon, wholly disproportioned to their ability, and which is to cause many a painful sacrifice and self-denial for weary weeks and months to come. The friends of the bride make their presents to show their appreciation of her; the friends of the bridegroom must do the same out of respect to the beautiful being who is so soon to become as one of their friends; the result is, that the young couple begin their married life with an amount of household stuff useful and ornamental, equal sometimes to many thousands of dollars, more or less of which is contributed by persons wholly unable to meet the expenditures; but did not wish to be behind others for fear of giving offence. This really seems to be a new method of levying black mail, which aforetime used to be considered one of the meanest ways ever devised for raising money. In order to goad the unwilling and unable, to contribute to the very utmost of their ability, the presentors are expected to put their names on the articles contributed, and it has even been said that, in some cases, the cost of each article is affixed to it. These things ought not to be. Let those who are starting out in life, stand on their own bottom; if they start upon the race on an even footing with others, and win their way by the power of their own right arm, then they will have the proud consciousness through life, that they have made themselves what they are, and that they owe their success wholly to themselves. Such a feeling is, of itself, worth a small fortune, and is more enduring, because it may be pleasureably drawn upon without diminution, to the end of life.

